# Note on the Dressing of Blisters

**Published:** 1848-11

**Authors:** Douglass Maclagan


*Note on the Dressing of Blisters.* By Douglass Maclagan, M. D. —I have for some years been in the habit of dressing blisters in a way not, I believe, generally adopted, but which I am induced to bring under the notice of the profession, as I have found it very convenient in practice. It consists merely in substituting, as is done in burns, dry cotton wadding for the ordinary dressing with healing ointment. When I order a blister, I generally direct that after it has been applied for the requisite number of hours, it shall be removed, and the part covered for two hours with a soft warm poultice of bread and milk. The effect of the poultice generally is to make the vesication more complete, and at the same time to moderate the tenderness of the blistered part. When the poultice is taken off, the vesication, if it has not burst spontaneously, is to be cut so as to discharge the serum, and then a thick layer of soft cotton wadding applied over the part with the undressed or woolly surface next the skin. If in the course of a few hours this should become soaked with the serous discharge from the blister, so much of the cotton may be removed as can be done without disturbing the loose epidermis beneath, and the whole again covered with a dry layer of cotton. This is all the dressing which is in general requisite. The cotton is allowed to stick to the skin of the blistered part, and when a fresh layer of epidermis is formed, which takes place very readily, the old epidermis and cotton come off together, leaving a smooth whole surface below.

The advantages which I have found this plan to offer are—first, that it renders the blister much less painful and annoying to the patient than when unguents are used. The tenderness in fact is comparatively so trifling, and the protection by the cotton so good, that I have been enabled without annoyance to the patient to percuss freely, and apply the stethoscope firmly over blistered parts, which had been dressed but an hour or two previously; secondly, the blisters heal faster under it than under dressings with cerate; for, although the cotton may remain adhering for some days, I have generally found that within twelve hours the patient ceases to feel the blister a source of annoyance. Lastly, it dispenses with the greasy applications so disagreeable to patients of cleanly habits.

The above plan has appeared to me particularly serviceable in an application of blisters which I am frequently in the habit of making—viz., to the treatment of ringworm. When this troublesome disease exists on the scalp in one or two detached spots, and more especially when it appears on the face, neck, or arms, it may, if taken in time, generally be cured in a few days, and prevented from spreading by blistering the surface *thoroughly*, and dressing the blisters with the cotton. A small cantharides plaster a few lines larger than the affected spot is much more efficient and manageable than the acetic acid, nitrate of silver, and other caustics frequently in use. I have seen very ugly sores formed on the neck of delicate females where caustic had been applied to the eruption, but I have never seen any bad effects follow the application of the blister, and the disease on this part of the body is generally cured by one application. I have seen it, however, fail on the scalp from this part, being less easy to vesicate thoroughly. Unless the whole affected surface is completely blistered, the disease is not cured, and therefore, where the eruption has been allowed to spread over a great part of the scalp, the blistering plan will not succeed, and would constitute a very severe and formidable plan of treatment,

Where, instead of wishing to heal a blister soon, the object of the practitioner is to keep it open, as in the endermic application of remedies, there is occasionally some difficulty experienced in maintaining a free raw surface. A little management will accomplish this. The blister should be somewhat larger than the surface which it is intended to keep open. When it has been kept on for the requisite time, the part is to be poulticed, especially if it has not risen freely. The whole loose epidermis is then to be removed, which a steady hand with a pair of forceps and scissors can accomplish, without causing more pain than follows the mere exposure of the raw surface to the air.

When a medicinal substance is to be applied, it is to be sprinkled over the raw surface, and the part covered for about two hours with a pledget of simple ointment. At the end of this time savin cerate is substituted for the simple dressing, and is to be maintained until the time when a second dose of the medicine is to be applied. The simple ointment is again laid on as before, and again alternated with the savin cerate.

By following this method, I have obtained satisfactory results from the endermic application of strychnia in neuralgia, and although I have applied the blister on the cheek in females, I have not found that it left any permanent mark on the part. I have generally used two grains of strychnia diluted with six or eight grains of white sugar, and divided into twelve powders, one of which may be applied night and morning. In several cases of severe neuralgia of some years standing, I have succeeded in giving great relief to the patients. In three instances, females of different ages, exemption from paroxysms of pain lasted between two and three months. I cannot say that I have been able to trace a radical cure directly to the strychnia, but it has appeared to me that the exemption from suffering for so long a period, has enabled them by out of door exercise and other means, to obtain an improvement of the general health, which has been followed by the ultimate removal of the disease.—*Edinburgh Monthly Journal of Med. Science.*

